# Validity and reliability of the Kinovea program in obtaining angles and distances using coordinates in 4 perspectives

**DOI:** 10.1371/journal.pone.0216448

**Published:** 2019-06-05

**Authors:** Albert Puig-Diví, Carles Escalona-Marfil, Josep Maria Padullés-Riu, Albert Busquets, Xavier Padullés-Chando, Daniel Marcos-Ruiz

**Affiliations:** 1 Blanquerna School of Health Sciences—Ramon Llull University, Barcelona, Catalunya, Spain; 2 Facultat de Ciències de la Salut de Manresa, Universitat de Vic–Universitat Central de Catalunya, Manresa (Barcelona), Catalunya, Spain; 3 Department of Physical Therapy, EUSES–University of Girona, Salt (Girona), Catalunya, Spain; 4 National Institute of Physical Education of Catalonia INEFC, Barcelona, Catalunya, Spain; Universidad Autonoma de Madrid, SPAIN

## Abstract

An objective analysis of the human movement can help both clinical assessment and sports performance. Kinovea is a free 2D motion analysis software that can be used to measure kinematic parameters. This low-cost technology has been used in sports sciences, as well as in the clinical and research fields. One interesting tool is that it can measure an object (or person) passing in front of the camera, taking into account the perspective between the camera and the recorded object. Although it has been validated as a tool to assess time-related variables, few studies assessed its validity compared to a Gold Standard; furthermore, its reliability in different perspectives has not been previously assessed. The main objective of this study is to determine the validity of the Kinovea software compared to AutoCAD, and its intra and inter-rater reliability in obtaining coordinates data; a second objective is to compare their results at 4 different perspectives (90°, 75°, 60° and 45°) and to assess the inter and intra rater reliability at each perspective. For this purpose, a wire structure figure in the shape of a human lower limb was designed and measured in AutoCAD; it was then recorded during a pendular motion with a video-camera placed at distance of 5 m and analyzed with Kinovea in the 4 perspectives (90°, 75°, 60° and 45°). Each frame was examined by three observers who made two attempts. A multiple approach was applied involving the analysis of the systematic error, with a two-way ANOVA 2x4; the relative reliability with Intraclass Correlation Coefficient (ICC) and the Coefficient of Variance (CV) (95% confidence interval); and the absolute reliability with the Standard Error (SE). The results indicate that the Kinovea software is a valid and reliable tool that is able to measure accurately at distances up to 5 m from the object and at an angle range of 90°–45°. Nevertheless, for optimum results an angle of 90° is suggested.

## Introduction

The study of kinematics is required given the need to objectify human movement in various fields, including sports management analysis, clinical research, footwear, and orthopedics [1], in order to obtain quantifiable data and to compare different subjects or different moments (pre and post treatment, training, etc.).

One of the most rigorous and scientifically validated systems used in kinematic analysis is the three-dimensional (3D) motion analysis laboratory, which provide very accurate data. However, it involves technical difficulties in interpretation and set-up [2], and high-cost instrumentation and programs, which can limit its use in research and in clinics [3]. New 2D low-cost technologies are nowadays available, some of which may have a precision comparable to leading high-end reference systems [4], with a significantly lower cost (approximately £700 -€950 according to Ugbolue et al. [5]. Prior to their standardized use to assess human gait (for example), it is important that these tools have proven to be valid and reliable.

One such low-cost technology is Kinovea, a free 2D motion analysis software under GPLv2 license, created in 2009 via the non-profit collaboration of several researchers, athletes, coaches and programmers from all over the world.

It enables the analysis of distances, angles, coordinates and spatial-temporal parameters [6] frame by frame from a video recording. These measurements can be made from different perspectives, since the software carries out calibrations in non-perpendicular planes to the camera-object line analyzed.

Kinovea has been used in three main fields: sports [7–18], clinical analysis [19–25], and as a tool with which to compare the reliability of other new technologies [26].

The 2D/3D program AutoCAD-2010 is commonly used in industrial design and architecture, and with scientific rigor in both the biomedical and engineering fields. Several AutoCAD applications have been described as tools for application in clinical and sports sciences [27–30] as a tool to measure distances, angles and coordinates.

Kinovea is an easy-to-use, portable and free tool that can be used in real field situations; no previous experience is required to obtain accurate and reliable measurements [6]. It has previously been validated as a tool to assess time-related variables [6]. Previous studies have inconclusive results: the reliability found ranges from poor to excellent, and the importance of the set-up has been highlighted [7–9, 31]. However, no analysis have been made regarding its reliability and validity in a controlled laboratory set-up and compared to AutoCAD software using a coordinates selection; furthermore, no analysis has been made regarding its reliability in measuring at different perspectives.

The objective of this study is two-fold: 1) to determine the validity of the Kinovea software comparing the coordinates obtained with Kinovea and AutoCAD -as a Gold Standard- in a moving cardboard that replicates a human lower limb during gait, and to determine its intra and inter rater reliability from an orthogonal perspective; 2) to compare its results at 4 different perspectives of 90°, 75°, 60° and 45° and to assess the inter and intra rater reliability at each perspective.

## Materials and methods

A prospective observational study was designed. A laboratory setting was used.

### Procedure

The Kinovea version analyzed was 0.8.24. The procedure included seven steps: 1. Design of a geometric figure; 2. Configuration and instrumentation of the recording space; 3. Image capture procedure; 4. Kinovea frame calibration; 5. Images digitization; 6. Export of data to spreadsheet; 7. Data extraction and transformation.

#### 1. Design of a geometric figure

The item to be measured using Kinovea was a purposely made geometric figure, designed using the AutoCAD-2010 program. Based on the Helen Hayes protocol [32], a lower limb was drawn as a wire structure simulating 5 moments of the human gait cycle, and 25 markers were placed on it at visible bony prominences [33]. These markers were numbered to help to establish an order during the analysis process. Four extra markers were drawn at the edges of the geometric template, forming an 800x555 mm rectangle. This rectangle was used as a reference to calibrate the frame with Kinovea.

Markers were drawn as black unfilled circles with a 25 mm outer diameter and a 2 mm inner diameter (i.e., solid white circles) in order to improve the precision of locating geometric centers in Kinovea [4, 5, 34].

**Fig 1:**
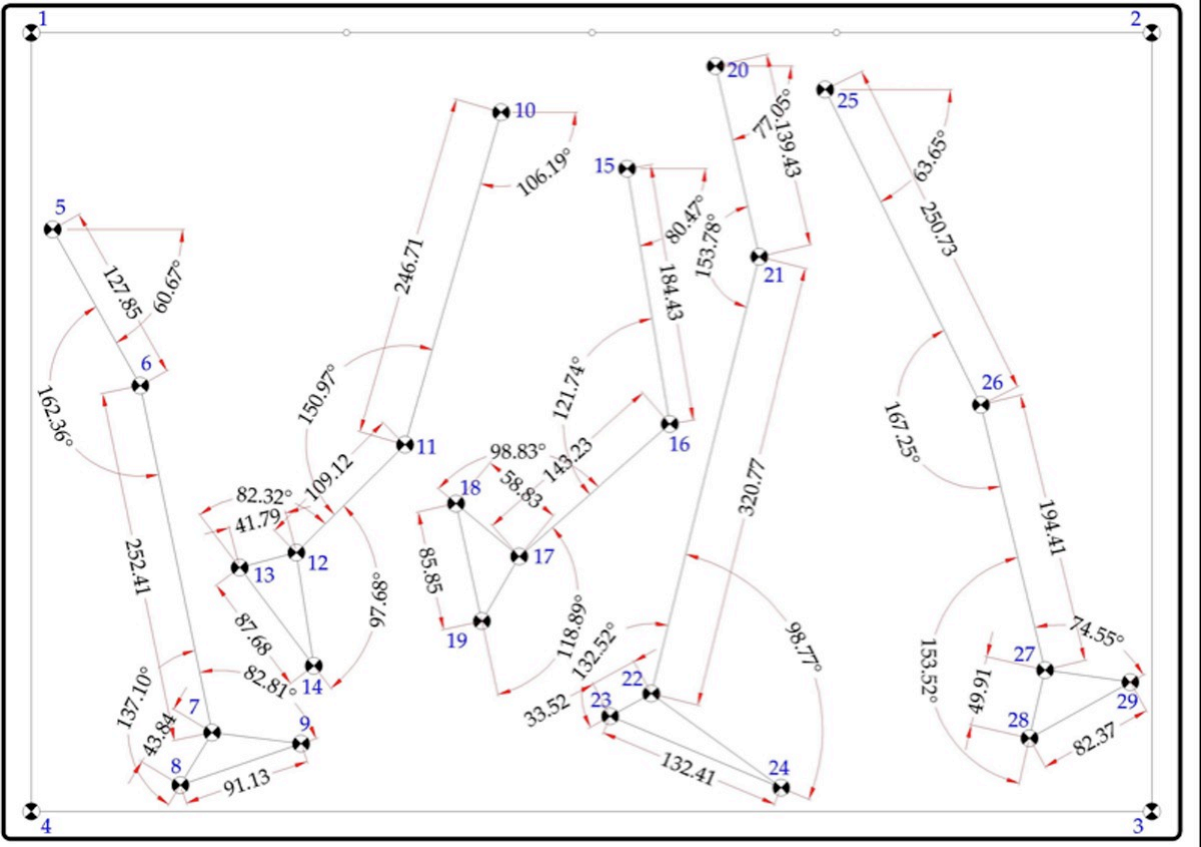
Geometric figure: Wire structure simulating lower limb during gait with the coordinates for each marker using AutoCad.

Each one of the 29 points were dimensioned in AutoCAD to obtain their coordinates in the x and y axis (Fig 1). These coordinates were exported to a spreadsheet and used to create trigonometric formulae that enabled the transformation of coordinates exported from Kinovea into distances and angles.

The geometric figure was then printed on an ISO-DIN-A1 paper using an HP Design jet T120 plotter and stuck to a 10 mm thick cardboard base. To check the accuracy of the printing and to confirm that the printed figure was scaled 1:1, three randomly selected distances (10%) between the markers were verified using a caliper.

#### 2. Configuration and instrumentation of the recording space

**Fig 2:**
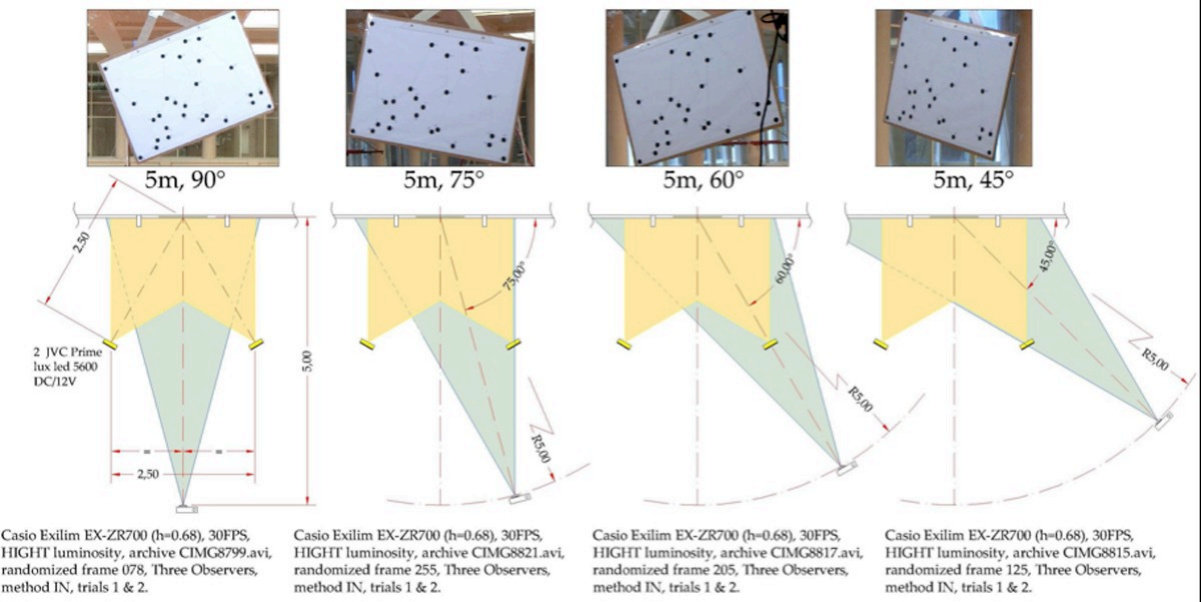
Setting: Setting used to record in the 4 perspectives.

The geometric figure was hung on a glass surface by means of a Ø115 mm Silverline suction cup, which incorporates a Ø4 mm rotation axis made of AISI-304 material on a self-lubricating nylon base to promote slippage. The geometric figure was recorded using a CASIO Exilim EX-ZR700 high definition video camera with the following setup: resolution: 1280x720 pixels per inch; frequency: 30 Hz; focal length: 52 mm; sensitivity: ISO 400; aperture: 2.7; and shutter time: 1.80. The lens was located at a height of 0.68 m from the ground and 5 m from the center of the recorded figure (Fig 2).

In order to precisely place the instrumentation, distances were measured with a BOSCH PR 15 laser distance measurer. The camera was placed perpendicular (90 degree) to the ground using a 40 cm magnetic bubble level (STANLEY ANTICHOC). Capture area illumination was achieved with two JVC Prime DC / 12V non-flickering LED lights.

The total recording area was 2.52x2.30 m (2520.35x2301.22 mm). Recordings were carried out from 4 different perspectives between the geometric figure and the camera: orthogonal (90°), 75°, 60°, and 45°; the same set-up was used for each of the four perspectives (Fig 2).

#### 3. Image capture procedure

**Fig 3:**
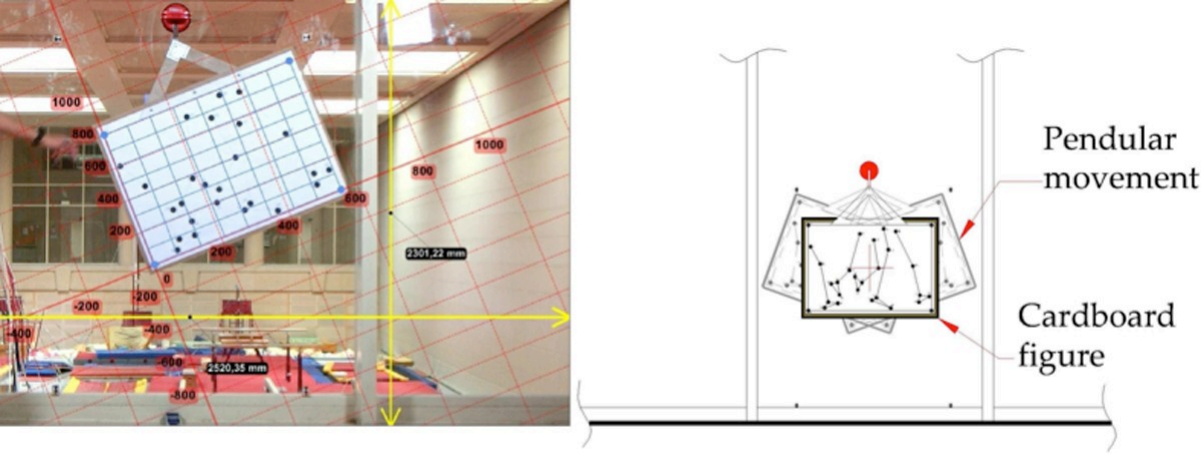
Setting and recording: Laboratory setting where the cardboard was recorded during the pendular movement.

The figure was pulled to one side by one researcher, the recording started, and the figure was released, causing a pendular movement (Fig 3). After a few seconds, when the figure had ceased moving, recording was stopped. This procedure was repeated for each of the four perspectives. One frame of the video files obtained from each perspective was randomly selected and analyzed using Kinovea, totaling 4 frames analyzed.

#### 4. Kinovea frame calibration

Each frame was analyzed twice by each of three observers. Three computers with the Kinovea program installed were used, including one desktop computer with a screen resolution and size of 1440x900 pixels and 48.37 cm (19.04 inches), respectively, and two notebooks with a screen resolution and size of 1366x768 pixels and 46.19 cm (18.18 inches), respectively. The entire image calibration and digitization process were completed using Logitech M305 wireless mouse.

The four selected frames were calibrated based on perspective via the <<perspective grid>> command, setting the corners of the grid at reference markers 1–4.

To improve the accuracy of grid placement, a 600% zoom was carried out and the grid ends placed on the geometric center of the markers via the <<scroll>> command.

Finally, the geometric reference system was calibrated based on the known dimensions between points 1, 2, 3 and 4 via the <<calibration>> command.

#### 5. Image digitization

**Fig 4:**
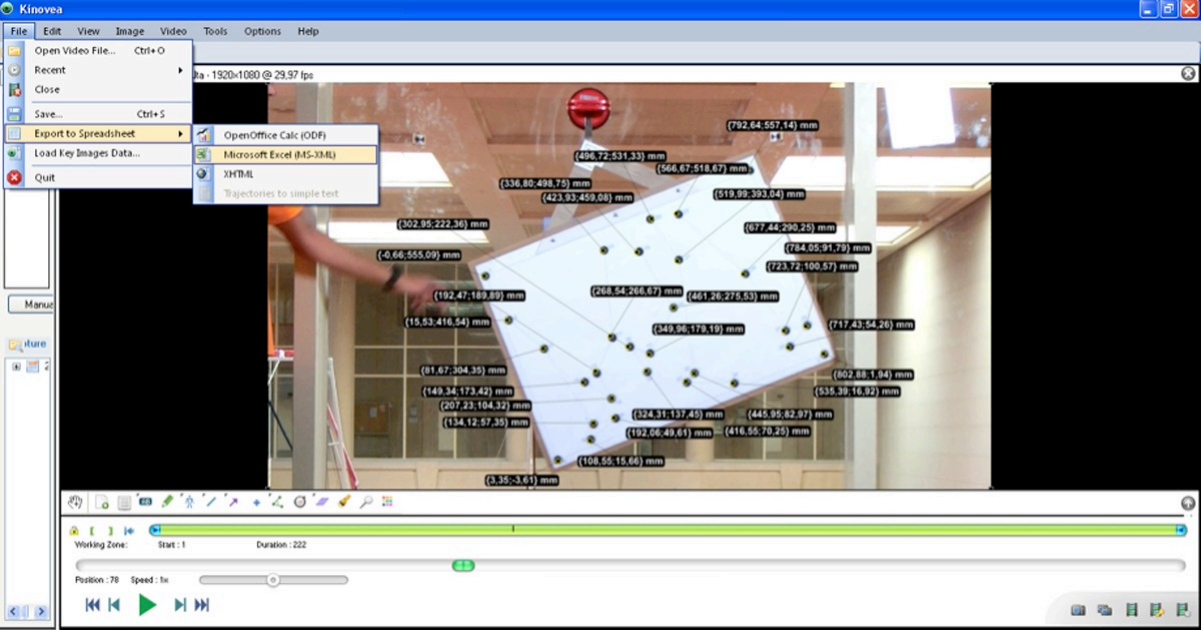
Kinovea coordinates: Coordinates were digitized in x-y axis using the Kinovea program.

For each one of the four frames, the twenty-nine points were digitized in their x and y coordinates on the geometric figure via the <<markers>> command. To improve digitization accuracy, the points were re-centered on the geometric center of the markers via the <<move>> command at an increased zoom (600%), with the coordinates of each point then displayed via the <<display coordinates>> command (Fig 4). The entire analysis procedure in Kinovea was carried out in each of the four perspectives (90°, 75°, 60°, and 45°) by the three observers. Each observer performed their analysis independently in different computers, time, and places, and they were blinded to one another’s results. Two trials or attempts were conducted on non-consecutive days by each observer, resulting in a total of 24 frames being analyzed (696 points digitized).

#### 6. Export data to spreadsheet

Once the points had been placed and their coordinates displayed, they were exported using the command <<Export to spreadsheet>>.

#### 7. Data extraction and transformation

The exported data were pasted into a spreadsheet containing trigonometric formulae that enabled the calculation of 20 angles and 20 distances between the points, based on their x-y coordinates.

Statistical analysis was performed using Microsoft Excel-2007 and PASW Statistics 18.0.

### Data sampling and analysis

The Shapiro-Wilks test was administered to determine data normality. The heteroscedasticity of the variables was calculated using Pearson's correlations between the average of the two trials and the absolute difference between the two trials. Heteroscedasticity test failed when positive and significant is found, that is, when higher values presented more variability than the lower values [35]. When normality and / or heteroscedasticity were not fulfilled, transformations were applied.

In order to evaluate the reliability and validity of the use of Kinovea from each of the four perspectives, and following the proposal made by several earlier authors [36–41], a multiple approach was applied involving tests of systematic error as well as relative and absolute reliability.

Systematic error (SE) between the two attempts and the four different perspectives was calculated for each observer via a two-way ANOVA [2 (Trial) x 4 (Perspective)]. When the assumption of sphericity was not fulfilled, the Greenhouse-Geisser correction was applied. In the event of a significant effect, post-hoc analysis was carried out with the Bonferroni correction. A low SE and good reliability can be inferred when there are no significant differences between attempts and/or planes.

Relative reliability reports consistency when making several attempts. Here relative reliability was assessed via the Intraclass Correlation Index (ICC) and the Coefficient of Variance (CV). The CV was calculated by dividing the Standard Deviation (SD) by the mean (M) and expressed as a percentage (%), with a good relative reliability defined by low CV values (close to 0) and high ICC values (≥0.80). The range of values within the specific probability including "true" reliability was calculated using the 95% Confidence Interval (CI) of CV and ICC.

Absolute reliability was examined using the Standard Error of Measurement (SEM), which expresses the variation between attempts, and the Minimum Detectable Change (MDC), which expresses the minimum change that must occur to be considered as a true change. The MDC percentage (MDC%) was also calculated to facilitate comparison between measurements.

Finally, data validity was tested using different Pearson correlations between the values of the coordinates obtained in the Kinovea software and the coordinates obtained by AutoCAD (gold standard).

## Results

### Data analysis

Error of the measure is considered as the difference between the coordinates measured with Kinovea and with AutoCAD (Gold Standard). Table 1 displays error, including mean and standard deviation for each observer, trial and perspective. Data on both axes (transformed when necessary) showed a normal distribution. Low levels of heteroscedasticity are consequence of the low correlation between the average of the two trials, and the absolute value of the difference between the trials (Table 2). Only the coordinates in x-axis from Observer 1 in the 45° perspective failed to pass heteroscedasticity test.

**Table 1:** Errors. Mean and SD of the absolute value of the difference between the coordinates measurement and the actual coordinates.

	Trial1 error(mm)		Trial2 error(mm)	
	Mean	SD	Mean	SD
**Coordinates in x-axis**				
*Observer1*				
45°	6.03	3.22	6.73	3.55
60°	2.67	1.36	3.17	1.55
75°	3.85	1.80	3.73	1.75
90°	2.41	1.34	1.32	1.01
*Observer2*				
45°	5.65	3.02	5.73	3.16
60°	2.28	1.37	2.02	1.19
75°	3.06	1.53	2.98	1.57
90°	1.46	1.04	1.58	1.13
*Observer3*				
45°	7.07	3.71	0.98	1.10
60°	2.41	1.34	2.75	1.21
75°	3.66	1.94	3.26	1.73
90°	0.79	0.92	0.98	1.10
**Coordinates in y-axis**				
*Observer1*				
45°	1.03	0.74	0.95	0.78
60°	1.34	0.91	1.50	0.86
75°	0.58	0.78	0.75	0.67
90°	1.04	0.83	1.02	1.09
*Observer2*				
45°	1.13	0.66	1.01	0.64
60°	1.13	0.63	1.17	0.71
75°	0.51	0.65	0.51	0.67
90°	0.77	0.71	0.55	0.49
*Observer3*				
45°	0.85	0.64	0.82	0.61
60°	1.04	0.83	1.33	0.84
75°	0.63	0.70	0.54	0.53
90°	0.61	0.44	0.82	0.61

**Table 2:** Mean (M) and standard deviation (SD) of the measured coordinates calculated for each perspective, and the absolute differences between the two trials.

		Trial 1 (mm)		Trial 2 (mm)		| Diff Trial 1-Trial 2 (mm) |			
		M	SD	M	SD	M	SD	rTrial1-Trial2	pTrial1-Trial2
	**Coordinates in x-axis**								
*Observer 1*									
	45 deg	373.07	252.74	372.03	252.40	1.17	0.63	0.360	0.029
	60 deg	376.36	252.20	376.08	246.83	0.96	0.60	-0.046	0.407
	75 deg	375.12	252.27	375.28	251.71	0.66	0.56	-0.456	0.006
	90 deg	376.61	251.82	379.83	251.59	3.30	1.77	-0.169	0.191
*Observer 2*									
	45 deg	373.57	252.72	373.20	252.46	0.53	0.50	0.260	0.086
	60 deg	376.85	252.27	377.27	252.21	0.55	0.42	0.288	0.065
	75 deg	376.01	252.20	376.04	252.11	0.37	0.30	-0.040	0.419
	90 deg	380.00	251.67	380.22	251.46	0.50	0.32	-0.191	0.160
*Observer 3*									
	45 deg	371.77	252.91	379.52	251.49	7.79	4.10	-0.357	0.029
	60 deg	376.61	251.82	376.21	252.19	0.77	0.88	0.186	0.166
	75 deg	375.25	252.07	375.80	252.17	0.60	0.38	0.171	0.187
	90 deg	379.35	251.52	379.52	251.49	0.63	0.44	0.154	0.212
**Coordinates in y-axis**									
*Observer 1*									
	45 deg ^a^	226.53	187.73	227.03	187.26	0.95	0.78	-0.041	0.416
	60 deg ^a^	228.33	187.58	228.47	183.41	0.81	0.60	-0.059	0.380
	75 deg ^a^	227.30	187.40	227.62	188.00	0.85	0.68	0.121	0.266
	90 deg ^a^	228.15	187.58	228.00	187.18	0.81	0.65	0.089	0.323
*Observer 2*									
	45 deg ^a^	226.34	186.79	226.49	187.74	0.51	0.35	0.121	0.266
	60 deg ^a^	228.20	185.99	228.21	187.74	0.41	0.32	-0.238	0.107
	75 deg ^a^	227.59	185.54	227.56	187.56	0.34	0.21	0.121	0.265
	90 deg ^a^	227.63	187.54	227.19	187.90	0.83	0.48	-0.386	0.019
*Observer 3*									
	45 deg ^a^	226.59	187.78	227.84	187.45	1.28	0.90	-0.253	0.093
	60 deg ^a^	228.15	187.58	228.44	187.39	0.64	0.56	-0.544	0.001
	75 deg ^a^	226.98	187.28	227.10	187.50	0.40	0.38	-0.083	0.335
	90 deg ^a^	227.48	187.79	227.84	187.49	0.77	0.50	0.016	0.468

Heteroscedasticity assessment is presented as the correlation between the mean of the two trials and their absolute difference.

^a^ data square-root transformed to assess correlation after normality test failure

The systematic error (SE) produced by the different observers was evaluated via ANOVA [2 (Trial) x 4 (Perspective)]. On the x-axis, an interaction (Trial x Perspective) was observed for observers 2 and 3, with a main effect on Trial for observer 3 (Table 3). Post-hoc analyses showed that the coordinates recorded from a 45° perspective differed between attempts for all observers.

**Table 3:** Two-way ANOVA RM comparing trials and perspectives conducted by the same observer.

		F	df	p	η^2^p	Power	Post-hoc
**Coordinates in x-axis**							
*Observer 1* ^a^							
	Trial x Perspective	0.557	3.112	0.645	0.015	0.16	-
	Perspective	0.444	3.112	0.722	0.012	0.14	-
	Trial	0.035	1.112	0.852	0.000	0.06	-
*Observer 2*							
	Trial x Perspective	9.474	3.112	0.001	0.202	1.00	45°: T1 >T2; 60°: T1 < T2
	Perspective	0.004	3.112	1.000	0.000	0.05	-
	Trial	0.925	1.112	0.338	0.008	0.16	-
*Observer 3*							
	Trial x Perspective	87.859	3.112	0.001	0.702	1.00	45°: T1 < T2
	Perspective	0.002	3.112	1.000	0.000	0.05	-
	Trial	97.290	1.112	0.001	0.465	1.00	T1 < T2
**Coordinates in y-axis**							
*Observer 1*							
	Trial x Perspective	1.996	3.112	0.119	0.051	0.50	-
	Perspective	0.000	3.112	1.000	0.000	0.05	-
	Trial	4.407	1.112	0.038	0.038	0.55	T1 < T2
*Observer 2*							
	Trial x Perspective	4.788	3.112	0.004	0.114	0.89	90°: T1 > T2
	Perspective	0.000	3.112	1.000	0.000	0.05	-
	Trial	1.788	1.112	0.184	0.016	0.26	-
*Observer 3*							
	Trial x Perspective	11.861	3.112	0.001	0.241	1.00	45°: T1 < T2; 90°: T1 < T2
	Perspective	0.000	3.112	1.000	0.000	0.05	-
	Trial	46.680	1.112	0.001	0.294	1.00	T1 < T2

^a^ Data transformed using natural logarithm to conduct ANOVA because heteroscedasticity test failed.

Intra-rater analysis revealed significant differences between the two trials conducted by observer 2 from a 60° perspective. The main effect found on Trial for observer 3 indicated differences between the attempts.

On the y-axis, an interaction (Trial x Perspective) was recorded in the trials conducted by observers 2 and 3, and a main effect on Trial for observers 1 and 3. Post-hoc analyses revealed significant differences between trials for observers 2 and 3 from a 90° perspective, but only for observer 3 from a 45° perspective.

The trials conducted by observers 1 and 3 were overall significantly different, with the sizes of the effect of the different interactions and the simple effects moderate or large (Table 3).

The relative reliability (ICC and CV) and absolute reliability (SEM and MDC) values are displayed in Table 4. The trials conducted from all perspectives by all observers presented very high values of ICC, indicating the reliability of the different observers. The ICC confidence interval (95%) values between 0.99 and 1 also provided greater data robustness.

**Table 4:** Inter-trial reliability of the three observers for each perspective.

		Mean(mm)	SD(mm)	Typical Error (mm)	ICC (95%CI)	SEM	CV (95% CI)	MDC	MDC (%)
**Coordinates in x-axis**									
*Observer 1*									
	45 deg ^a^	372.55	250.34	0.82	0.99 (0.99–1.00)	0.07	10.34 (10.05–10.65)	0.19	0.03
	60 deg	376.22	250.26	0.68	1.00 (1.00–1.00)	0.00	-6.21 (-6.46 - (-5.97)	0.00	0.00
	75 deg	375.19	249.77	0.47	1.00 (1.00–1.00)	0.00	NaN	0.00	0.00
	90 deg	378.22	249.5	2.36	1.00 (1.00–1.00)	0.00	-0.04 (-0.89 - (-0.82)	0.00	0.00
*Observer 2*									
	45 deg	373.39	250.36	0.37	1.00 (1.00–1.00)	0.00	3.95 (3.81–4.08)	0.00	0.00
	60 deg	377.06	250.02	0.39	1.00 (1.00–1.00)	0.00	5.84 (5.70–5.99)	0.00	0.00
	75 deg	376.02	249.93	0.26	1.00 (1.00–1.00)	0.00	13.23 (13.14–13.32)	0.00	0.00
	90 deg	380.16	249.35	0.35	1.00 (1.00–1.00)	0.00	NaN	0.00	0.00
*Observer 3*									
	45 deg	375.64	250.01	5.51	1.00 (1.00–1.00)	0.00	-15.68 (-17.68 - (-13.67)	0.00	0.00
	60 deg	376.41	249.79	0.54	1.00 (1.00–1.00)	0.00	-15.11 (-15.30 - (-14.91)	0.00	0.00
	75 deg	375.53	249.90	0.42	1.00 (1.00–1.00)	0.00	5.42 (5.27–5.58)	0.00	0.00
	90 deg	379.43	249.29	0.45	1.00 (1.00–1.00)	0.00	-8.25 (-8.41 - (-8.09)	0.00	0.00
**Coordinates in y-axis**									
*Observer 1*									
	45 deg	226.78	185.84	0.67	1.00 (1.00–1.00)	0.00	7.57 (7.33–7.82)	0.00	0.00
	60 deg	228.40	185,72	0,57	1.00 (1.00–1.00)	0,00	-160.18 (-160.39 - (-159.98)	0,00	0,00
	75 deg	227,46	186,05	0,60	1.00 (1.00–1.00)	0,00	-149.87 (-150.09 - (-149.65)	0,00	0,00
	90 deg	228,08	185,73	0,57	1.00 (1.00–1.00)	0,00	-0.40 (-0.60 - (-0.19))	0,00	0,00
*Observer 2*									
	45 deg	226.41	186.11	0.36	1.00 (1.00–1.00)	0.00	138.83 (130.70–138.96)	0.00	0.00
	60 deg	228.20	185.99	0.28	1.00 (1.00–1.00)	0.00	-47.50 (-47.61 - (-47.40)	0.00	0.00
	75 deg	227.58	185.89	0.24	1.00 (1.00–1.00)	0.00	2.28 (2.19–2.37)	0.00	0.00
	90 deg	227.41	186.06	0.59	1.00 (1.00–1.00)	0.00	NaN	0.00	0.00
*Observer 3*									
	45 deg	227.21	185.98	0.91	1.00 (1.00–1.00)	0.00	8.64 (8.63–8.97)	0.00	0.00
	60 deg	228.29	185.83	0.54	1.00 (1.00–1.00)	0.00	-0.10 (-0.26–0.07)	0.00	0.00
	75 deg	227.04	185.74	0.28	1.00 (1.00–1.00)	0.00	2.75 (2.65–2.85)	0.00	0.00
	90 deg	227.67	185.98	0.54	1.00 (1.00–1.00)	0.00	2.90 (2.70–3.09)	0.00	0.00

^a^ Data transformed using natural logarithm to conduct ICC, SEM, MDC, and MDC (%) because heteroscedasticity test failed.

Standardization of errors based on CV (%) revealed considerable dispersion of values, especially on the y-axis. The differences found between trials when digitizing the same marker affected the CV mean for the same perspective. Values of absolute reliability (SEM and MDC) were low, equal or very close to 0 (Table 4), which indicates low error for the different perspectives and observers.

In terms of data validity, Pearson coefficient values were very high regarding the correlation between the observations and the actual data (Table 5). The smallest typical error of the estimate (TEE) was always found for trials conducted from a 90° perspective, while the highest overall value was recorded from a 45° perspective. Nevertheless, the low TEE values demonstrate the overall validity of the data obtained using Kinovea.

**Table 5:** Correlation values among perspectives for each observer and for all observers pooled.

	Observer 1			Observer 2			Observer 3			Observers pooled		
	r	p	TEE	r	p	TEE	r	p	TEE	r	p	TEE
**Coordinates in x-axis**												
45 deg vs Real coordinates	1.00	0.001	3.671	1.00	0.001	3.595	1.00	0.001	1.979	1.00	0.001	3.043
60 deg vs Real coordinates	1.00	0.001	1.637	1.00	0.001	1.751	1.00	0.001	1.608	1.00	0.001	1.626
75 deg vs Real coordinates	1.00	0.001	2.25	1.00	0.001	2.020	1.00	0.001	2.242	1.00	0.001	2.157
90 deg vs Real coordinates	1.00	0.001	1.182	1.00	0.001	1.145	1.00	0.001	1.083	1.00	0.001	1.042
**Coordinates in y-axis**												
45 deg vs Real coordinates	1.00	0.001	1.026	1.00	0.001	0.998	1.00	0.001	0.697	1.00	0.001	0.847
60 deg vs Real coordinates	1.00	0.001	0.948	1.00	0.001	0.778	1.00	0.001	0.764	1.00	0.001	0.741
75 deg vs Real coordinates	1.00	0.001	0.777	1.00	0.001	0.675	1.00	0.001	0.749	1.00	0.001	0.664
90 deg vs Real coordinates	1.00	0.001	0.873	1.00	0.001	0.736	1.00	0.001	0.576	1.00	0.001	0.585

TEE: typical error of the estimate

### Data interpretation

The results of the reliability tests (SEM, MDC, ICC, and CV) support the precision of the protocol. SE values were small for both attempts and perspectives.

The validity assessment was then repeated, producing a result of ICC = 1. One reason for this is that SEM and MDC were calculated from ICC; these two measures are thus linked, which provides great reliability when expressed to two decimal places.

Data validity can be considered acceptable for all planes (perspectives), with a correlation value of 1 (ICC = 1) obtained for all three observers.

The digitized values of the 29 x-axis coordinates were used to calculate an average of the set of values obtained by means of ANOVA. Slight numerical differences in the values of the paired trials were detected, as well as in the mixture or combination of the attempts made from different perspectives by observer 1. However, no significant differences were recorded in TEE values.

In summary, the obtained statistics show that the numerical difference between attempts one and two were mathematically very small.

## Discussion

As previously stated in the literature review, Kinovea is widely used for human motion analysis in both sports and clinical sciences. Furthermore, it has also been validated as a time measurement tool [6], and as such is used as a reference method with which to compare new technologies based on temporal space analysis. However, to the best of this author’s knowledge, the present study is the first to assess the reliability and validity of Kinovea in measuring distances and angles from different perspectives based on a coordinate system.

Although there are other video graphic analysis programs available, such as Dartfish, which has been used previously for scientific research [42, 43], the latter does not allow the correction of perspective and cannot be considered a ‘low cost’ tool, since it is not free as Kinovea is.

The present study has also paid special attention to the data digitization protocol employed, which must be controlled so as to avoid potential biases when taking repeated measurements. We propose the use of black circular markers with a smaller white circle in the center, in an attempt to increase the repeatability of this step, as well as a 600% zoom view to set the marker centers.

To assess the validity and reliability of Kinovea, an 800x555 mm geometric figure was constructed, with 29 points simulating a lower limb in different positions of the gait cycle. The figure was suspended and pushed to create a pendular movement, which was recorded with a video camera located at a distance of 5 m and using a 2.52x2.30 m recording area. Four frames were selected, one from each perspective, and digitized in Kinovea. Three observers each made two attempts to digitize the images into coordinates; these coordinates were then exported to a spreadsheet to be transformed into angles and distances.

The reliability of Kinovea coordinate digitization has been assessed previously by other authors. The inter and intra-observer reliability found in the present study is slightly higher than that reported elsewhere, including the ICC value of >0.79 reported in [7], the ICC value of 0.997 obtained in [6], and the Kappa index value >0.80 reported in [9]. Furthermore, the Pearson correlation coefficients obtained in the present study indicate very high correlation between data.

The results show that Kinovea is reliable when measuring in the perspective range from 90° to 45° and at a 5 m distance from the registered object. However, the differences found between the four tested perspectives suggest that Kinovea is best employed at 90° rather than 45°.

Nevertheless, according to the reliability tests performed in this study (SEM, MDC, ICC and CV), Kinovea can be considered reliable when employed at any of the four perspectives analyzed. In addition, SE values were small for both attempts and perspectives.

The validity tests confirmed that the obtained results are acceptable for all perspectives, with a correlation value of 1 (ICC = 1) recorded for all three observers. One possible explanation for this is that SEM and MDC were calculated from ICC; these measures are thus linked, which provides great reliability at two decimal places.

In fact, the measurement accuracy typically required from a clinical or sports science point of view is not as high as that reported in this paper; whereas all measurements were here made at the millimeter scale, in clinical practice an accuracy down to the centimeter may be assumed [5].

However, some limitations should be pointed. To the author’s knowledge, this is the first study to compare 4 different perspectives; although it is a positive point, a laboratory setting was prepared, using a 2D printed cardboard and not a real subject.

Future research should involve an assessment of the reliability and validity of Kinovea as a 2D tool for real gait analysis, the use of a 3D system as a gold standard, as well as the development of a standard 2D laboratory set-up for clinical and sports science research.

## Conclusions

The results of this study suggest that Kinovea is a valid, precise and reliable (both inter- and intra-rater) program with which to obtain angles and distance data from coordinates. These data can be obtained valid and reliably in different perspectives, from 90 to 45 degree. However, an orthogonal perspective (90 degree) is recommended. Biomechanical measurements can be obtained under rigorous digitization, suitable for use in the scientific, clinical and sporting fields.

Kinovea is a free, reliable tool that produces valid data, providing an acceptable level of accuracy in angular and linear measurements obtained via digitization of x- and y-axis coordinates.

## Supporting information

S1 Fig Minimum data set. Data set used to perform the statistical analysis, and results.
(XLSX)

S2 Fig Short video in which the "Methods" section proceeding is summarized: Design, capture and record moving images, calibration, scanning, and data extraction and processing (also available through the following link: https://drive.google.com/open?id=0BzhpXyL0tZoCMmdMWVloVGxGM2c). (MP4)

## References

[1] Maas J, Dallmeijer A, Huijing P, Brunstrom-Hernandez J, van KP, Bolster E, et al A randomized controlled trial studying efficacy and tolerance of a knee-ankle-foot orthosis used to prevent equinus in children with spastic cerebral palsy. Clin Rehabil. 2014;28(10):1025–38. 10.1177/0269215514542355 25082956

[2] Krishnan C, Washabaugh EP, Seetharaman Y. A low cost real-time motion tracking approach using webcam technology. J Biomech. 2015;48(3):544–8. 10.1016/j.jbiomech.2014.11.048 25555306 PMC4306621

[3] Macleod CA, Conway BA, Allan DB, Galen SS. Development and validation of a low-cost, portable and wireless gait assessment tool. Med Eng Phys. 2014;36(4):541–6. 10.1016/j.medengphy.2013.11.011 24345892

[4] Thewlis D, Bishop C, Daniell N, Paul G. Next-generation low-cost motion capture systems can provide comparable spatial accuracy to high-end systems. JApplBiomech. 2013;29(1):112–7. 10.1123/jab.29.1.112 22813783

[5] Ugbolue UC, Papi E, Kaliarntas KT, Kerr A, Earl L, Pomeroy VM, et al The evaluation of an inexpensive, 2D, video based gait assessment system for clinical use. GaitPosture. 2013;38(3):483–9. 10.1016/j.gaitpost.2013.01.018 23465758

[6] Balsalobre-Fernandez C, Tejero-Gonzalez CM, del Campo-Vecino J, Bavaresco N. The concurrent validity and reliability of a low-cost, high-speed camera-based method for measuring the flight time of vertical jumps. J Strenght Cond Res. 2014;28(2):528–33. 10.1519/JSC.0b013e318299a52e 23689339

[7] Sañudo B, Rueda D, Pozo-Cruz BD, de Hoyo M, Carrasco L. Validation of a Video Analysis Software Package for Quantifying Movement Velocity in Resistance Exercises. J Strenght Cond Res. 2016;30(10):2934–41. 10.1519/JSC.0000000000000563 24918300

[8] Dias JA, Pupo JD, Reis DC, Borges L, Santos SG, Moro AR, et al Validity of Two Methods for Estimation of Vertical Jump Height. J Strenght Cond Res. 2011;25(7):2034–9. 10.1519/JSC.0b013e3181e73f6e 21701288

[9] Damsted C, Nielsen RO, Larsen LH. Reliability of video-based quantification of the knee- and hip angle at foot strike during running. Int J Sports Phys Ther. 2015;10(2):147–54. 25883863 PMC4387722

[10] Grigore V, Gavojdea AM, Potop V. Analysis on Biomechanical Characteristics of Dismounts in Salto Backward Stretched Landings, in Balance Beam Event. Icpesk. Bucharest, 2015 p. 125–30.

[11] Potop V, Timnea OC, Grigore V. E-learning and technology of transfer based on video computerized analysis of sports technique of acrobatic exercises on floor in women's artistic gymnastics In: Roceanu I, editor. Let's Build the Future through Learning Innovation! eLearning and Software for Education. Carol I Natl Defence Univ Publishing House: Bucharest 2014 p. 177–82.

[12] Potov V, Boloban V, Timnea OC. Analysis of Biomechanical Characteristics of Yurchenko Vault Sports Technique in Women's Artistic Gymnastics. Icpesk. Bucharest, 2015 p. 477–82.

[13] Ismail SI, Adnan R, Sulaiman N. Moderate Effort Instep Kick in Futsal. Procedia Eng. 2014;72:186–91.

[14] Torres-Luque G, Ramirez A, Cabello-Manrique D, Nikolaidis PT, Alvero-Cruz JR. Match analysis of elite players during paddle tennis competition. Int J Perform Anal Sport. 2015;15(3):1135–44.

[15] Torralba Jordán MA, Padullés Riu JM, Losada López JL, López del Amo JL. Alternativa ecológica en la evaluación del salto de longitud de atletas paralímpicos. Cuad Psicol del Deport. 2016;16:69–76.

[16] Rozan M, Rouhollahi V, Rastogi A, Dureha DK. Influence of physiological loading on the lumbar spine of national level athletes in different sports. J Hum Kinet. 2016;50(1):115–23. 10.1515/hukin-2015-0148 PMC5260646 28149348

[17] Aguilar LM, Torres JP, Jimenes CR, Cabrera DR, Cárdenas MF, Urgirles PF. Analysis of the angles in hip, knee and ankle during the pedaling of a Cross Country Olympic cyclist. Electron Eng Inf Commun Technol Proc: IEEE Chilecon; 2015 p. 205–8.

[18] Ramon Suarez G, Gaviria Alzate S, Teller Carreno DC, Calderon Rojas M, Ruiz Correa V. Evaluation scale of visual and auditive action-reaction times in youth karate athletes of Antioquia, Colombia. Viref Rev Educ Fis. 2016;5(1):1–16.

[19] Guzmán-Valdivia CH, Blanco-Ortega A, Oliver-Salazar MA, Carrera-Escobedo JL. Therapeutic Motion Analysis of Lower Limbs Using Kinovea. Int J Comput Eng. 2013;3(2):359–65.

[20] Damsted C, Larsen LH, Nielsen RO. Reliability of video-based identification of footstrike pattern and video time frame at initial contact in recreational runners. Gait Posture. 2015;42(1):32–5. 10.1016/j.gaitpost.2015.01.029 25920964

[21] Moral-Munoz JA, Esteban-Moreno B, Arroyo-Morales M, Cobo MJ, Herrera-Viedma E. Agreement Between Face-to-Face and Free Software Video Analysis for Assessing Hamstring Flexibility in Adolescents. J Strength Cond Res. 2015;29(9):2661–5. 10.1519/JSC.0000000000000896 26313580

[22] Candido PEF, Teixeira JVS, Moro ARP, Gontijo LA. Biomechanical strain of goldsmiths. Work-a Journal of Prevention Assessment & Rehabilitation. 2012;41(SUPPL. 1):2506–9. 10.3233/WOR-2012-0490-2506 22317096

[23] Rebolledo Uribe JP, Pincheira Barbé PA, Bittner Schmidt V, Frugone Zambra RE. Occlusal plane inclination in children between 9 and 10 years of age with postural asymmetry. A study on the frontal plane. Rev Fac Odontol Univ Antioquia. 2012;24(1):76–84.

[24] Hernández-Gervilla Ó, Escalona-Marfil C, Corbi F. Correlation between foot posture and running kinematics: a pilot study. Apunt Med l’Esport. 2016;51(192):115–22.

[25] Youssef AR. Photogrammetric quantification of forward head posture is side dependant in healthy participants and patients with mechanical neck pain. Int J Physiother. 2016;3(3):326–31.

[26] Padulo J, Vando S, Chamari K, Chaouachi A, Bagno D, Pizzolato F. Validity of the MarkWiiR for kinematic analysis during walking and running gaits. Biol Sport. 2015;32(1):53–8. 10.5604/20831862.1127282 25729150 PMC4314604

[27] Ramos-Ortega J, Dominguez G, Castillo JM, Fernandez-Seguin L, Munuera PV. Angular position of the cleat according to torsional parameters of the cyclist's lower limb. Clin J Sport Med. 2014;24(3):251–5. 10.1097/JSM.0000000000000027 24451688

[28] Elnahhas A, El-Negmy E, El-Azizi H. Calf Muscle Strength and Standing Efficiency in Children with Spastic Diplegia. Trends Appl Sci Res. 2014;9(9):503.

[29] Quieregatto PR, Hochman B, Furtado F, Machado AF, Sabino Neto M, Ferreira LM. Image analysis software versus direct anthropometry for breast measurements. Acta Cir Bras. 2014;29(10):688–95. 25318002 10.1590/s0102-8650201400160010

[30] Pontes LF, Cecim RL, Machado SM, Normando D. Tooth angulation and dental arch perimeter-the effect of orthodontic bracket prescription. Eur J Orthod. 2015;37(4):435–9. 10.1093/ejo/cju055 25316494

[31] Baude M, Hutin E, Gracies JM. A Bidimensional System of Facial Movement Analysis Conception and Reliability in Adults. Biomed Res Int. 2015;1:1–8. 10.1155/2015/812961 PMC4486482 26161415

[32] Mentiplay BF, Perraton LG, Bower KJ, Pua YH, McGaw R, Heywood S, et al Gait assessment using the Microsoft Xbox One Kinect: Concurrent validity and inter-day reliability of spatiotemporal and kinematic variables. J Biomech. 2015;48(10):2166–70. 10.1016/j.jbiomech.2015.05.021 26065332

[33] Northeast L, Gautrey CN, Bottoms L, Hughes G, Mitchell ACS, Greenhalgh A. Full gait cycle analysis of lower limb and trunk kinematics and muscle activations during walking in participants with and without ankle instability. Gait Posture. 2018;64:114–118. 10.1016/j.gaitpost.2018.06.001 29902713

[34] Vitorio R, Lirani-Silva E, Barbieri FA, Raile V, Batistela RA, Stella F, et al The role of vision in Parkinson's disease locomotion control: Free walking task. Gait Posture. 2012;35(2):175–9. 10.1016/j.gaitpost.2011.09.002 21962407

[35] Nuzzo JL, Anning JH, Scharfenberg JM. The reliability of three devices used for measuring vertical jump height. J Strength Cond Res. 2011;25(9):2580–5290.34. 10.1519/JSC.0b013e3181fee650 21804426

[36] Atkinson G, Nevill AM. Statistical methods for assessing measurement error (reliability) in variables relevant to sports medicine. Sports Med. 1998;26(4):217–38. 10.2165/00007256-199826040-00002 9820922

[37] Ditroilo M, Forte R, McKeown D, Boreham C, De Vito G. Intra-and inter-session reliability of vertical jump performance in healthy middle-aged and older men and women. J Sports Sci. 2011;29(15):1675–82. 10.1080/02640414.2011.614270 22098486

[38] Hopkins WG. Measures of reliability in sports medicine and science. Sports med. 2000;30(1):1–15. 10.2165/00007256-200030010-00001 10907753

[39] Webber SC, Porter MM. Reliability of ankle isometric, isotonic, and isokinetic strength and power testing in older women. Phys Ther. 2010;90(8):1165–75. 10.2522/ptj.20090394 20488976

[40] Weir JP. Quantifying test-retest reliability using the intraclass correlation coefficient and the SEM. J Strength Cond Res. 2005;19(1):231–40. 10.1519/15184.1 15705040

[41] Olper L, Cervi P, De Santi F, Meloni C, Gatti R. Validation of the treadmill six-minute walk test in people following cardiac surgery. Physical Ther. 2011;91(4):566. 10.2522/ptj.20100156 21310897

[42] Norris BS, Olson SL. Concurrent validity and reliability of two-dimensional video analysis of hip and knee joint motion during mechanical lifting. Physiother Theory Pract. 2011;27(7):521–30. 10.3109/09593985.2010.533745 21568816

[43] Eltoukhy M, Asfour S, Thompson C, Latta L. Evaluation of the performance of digital video analysis of human motion: Dartfish tracking system. Int J Sci Eng Res. 2012;3:1–6.

